# The role of CORM-2 as a modulator of oxidative stress and hemostatic parameters of human plasma *in vitro*

**DOI:** 10.1371/journal.pone.0184787

**Published:** 2017-09-26

**Authors:** Weronika Adach, Beata Olas

**Affiliations:** Department of General Biochemistry, Faculty of Biology and Environmental Protection, University of Lodz, Lodz, Poland; University of PECS Medical School, HUNGARY

## Abstract

**Purpose:**

The main aim of the experiment is to examine the effect of CORM-2, a donor of carbon monoxide (CO), on oxidative stress in human plasma *in vitro*. In addition, it examines the effects of CORM-2 on the hemostatic parameters of plasma: the activated partial thromboplastin time (APTT), thrombin time (TT) and prothrombin time (PT).

**Methods:**

Human plasma was incubated for 5–60 min with different concentrations of CORM-2: 0.1–100 μM. Following this, various hemostatic factors and biomarkers of oxidative stress were studied. Lipid peroxidation was measured as thiobarbituric acid reactive substance (TBARS) concentration, and the oxidation of amino acid residues in proteins was measured by determining the amounts of carbonyl and thiol groups.

**Results:**

Two oxidative stress inducers: hydrogen peroxide (H_2_O_2_) and the donor of hydroxyl radical (H_2_O_2_/Fe) were used. Decrease in protein carbonylation, thiol group oxidation and lipid peroxidation were detected at tested concentrations of CORM-2.

**Conclusion:**

Our results indicate that CORM-2 may have antioxidant properties in human plasma treated with H_2_O_2_ or H_2_O_2_/Fe. In addition, our results indicate the anti-coagulant activities of CORM-2 *in vitro*.

## Introduction

For decades, scientists have wondered if the biological relevance of toxic carbon monoxide (CO) could be isolated. The fact that uncontrolled quantities of inhaled gas lead to serious system complications and neuronal disturbances cannot be ignored. CO is a colorless, odorless and non-irritating gas. It is produced endogenously in the body, however, at elevated concentrations is highly toxic to organism [1]. During heme degradation, CO (by heme oxygenase (OH)) is synthetized. CO is also produced as a result of photooxidation, metabolism of xenobiotics and lipid peroxidation [2–5]. Nowadays, CO is becoming an important and versatile physiological mediator that has a beneficial effect on many models of vascular and inflammatory diseases [6–8]. CO regulates blood pressure and inhibits blood platelet aggregation [9–11]. Nielsen and Pretorius [12] suggest that CO may not only act as an anticoagulant, but also as a procoagulant. In addition, results of Nielsen and Garza et al. [13] demonstrated that CO—releasing molecules (CORMs): CORM-2 and CORM-3 enhanced thrombus strength and the velocity of clot formation in human plasma. Therefore, the question is whether there is a possibility to achieve therapeutic and safe concentrations of CO in tissues and organs, without providing a toxic dose through the lungs [1].

Because, mechanism(s) involved the relationship between the action of CO and various components of hemostasis (i.e. blood platelets, the coagulation and fibrinolysis) are still unknown, the aim of our experiments was to explain the effects of CORM-2 (the donor of CO; tricarbonyldichlororuthenium (II) dimer; Ru_2_Cl_4_(CO)_6_; at tested concentrations: 0.1–100 μM) on the three hemostatic parameters: the activated partial thromboplastin time (APTT), thrombin time (TT) and prothrombin time (PT) of human plasma *in vitro*. In addition, as the influence of donors of CO on oxidative stress remains also unknown, the aim of our experiment was to also determine the effects of CORM-2 on oxidative stress in human plasma when administered at concentrations of 0.1 to 100 μM *in vitro*. The study tests the activity of CORM-2 against the effect of a strong biological oxidant, either hydrogen peroxide (H_2_O_2_) or H_2_O_2_/Fe (a hydroxyl radical donor), on plasma lipids and proteins. Oxidative modifications of these components, especially plasma proteins are very important for modulating of hemostasis. They have been reported in various conditions, including cardiovascular disorders. Oxidative stress was measured by examining the concentrations of well-known oxidative biomarkers such as thiobarbituric acid reactive substance (TBARS), a marker of lipid peroxidation, the concentration of carbonyl groups and the concentration of thiol groups, which are markers of oxidative damage in proteins.

## Materials and methods

CORM-2, 5,5’-dithio-bis-(2-nitrobenzoic acid) (DTNB), thiobarbituric acid (TBA) and H_2_O_2_ were purchased from Sigma Chemical Co. (Germany). The coagulation reagents were obtained from Diagon Ltd. (Hungary) and Behringer Ingelheim. All other chemicals were reagent-grade products purchased from POCh (Gliwice, Poland).

A stock solution of CORM-2 was made in DMSO (the final concentration of DMSO in a stock solution of CORM-2 was 50%). The final concentration of DMSO in samples was lower than 0.05% and its effects were determined in all experiments. Inactive CORM-2 (i-CORM-2) was prepared by keeping the stock solution in 50% DMSO over night at 37°C [13,14].

### Exposure of human plasma to CORM-2

Fresh human plasma was obtained from medication-free, regular donors at the blood bank (Lodz, Poland). Samples of human plasma were exposed to 1) CORM-2 at a final concentration between 0.1 and 100 μM plus 2 mM H_2_O_2_; 2) CORM-2 at a final concentration between 0.1 and 100 μM plus 4.7 mM H_2_O_2_/3.8 mM Fe_2_SO_4_/2.5 mM EDTA. Samples were incubated for 5, 15, 30 and 60 minutes at 37°C. In addition, plasma was exposed to iCORM-2 (at a final concentration 100 μM) or CORM-2 (at a final concentration 100 μM) and albumin (at a final concentration 10%) for 30 minutes at 37°C. Moreover, samples of plasma were also exposed to iCORM-2 (at a final concentration 100 μM) or CORM-2 (at a final concentration 100 μM) and albumin (at a final concentration 10%) plus 4.7 mM H_2_O_2_/3.8 mM Fe_2_SO_4_/2.5 mM EDTA for 30 minutes at 37°C.

The protein concentration by measuring absorbance at 280 nm (in tested samples) was calculated according to the procedure of Whitaker and Granum [15].

### Lipid peroxidation measurement

Lipid peroxidation was quantified by measuring the concentration of TBARS. The incubation of plasma (controls, tested compounds) was stopped by cooling in an ice bath. The plasma samples were transferred to an equal volume of 15% (v/v) cold trichloroacetic acid in 0.25 M HCl and centrifuged at 8000 x g for 15 minutes. One volume of clear supernatant was mixed with 0.2 volume of 0.12 M thiobarbituric acid in 0.25 M HCl and immersed in a boiling water bath for 15 minutes. Then the cooled samples was centrifuged at 8000 x g for 5 min and the absorbance was measured at 535 nm using the SPECTROstar Nano Microplate Reader- BMG LABTECH (Germany) [16,17]. The TBARS concentration was calculated using the molar extinction coefficient (ε = 156,000 M^-1^cm^-1^). The level of TBARS (as nmol TBARS/ml of plasma) was expressed as % of control.

### Carbonyl group measurement

The detection of carbonyl groups in proteins was carried out according to Levine et al. [18] and Bartosz [17]. Carbonyl content was measured by absorbance at 375 nm (the SPECTROstar Nano Microplate Reader- BMG LABTECH, Germany). The carbonyl group concentration was calculated using a molar extinction coefficient (ε = 22,000 M^-1^cm^-1^), and the level of carbonyl groups (as nmol carbonyl groups/mg of plasma protein) was expressed as % of control.

### Thiol group measurement

The thiol group content in plasma proteins was measured spectrophotometrically (the SPECTROstar Nano Microplate Reader- BMG LABTECH, Germany) by absorbance at 412 nm with Ellman’s reagent. The thiol group concentration was calculated using the molar extinction coefficient (ε = 13,600 M^-1^cm^-1^) [17,19,20]. The level of thiol groups was expressed as μmol thiol groups/ml of plasma.

### The measurement of PT

Human plasma (50 μl) was incubated for 2 min. at 37°C on block heater. After incubation, the measuring cuvette was transferred to the measuring holes and 100 μl of Dia-PT liquid (commercial preparation) was added. The PT was determined coagulometrically (Optic Coagulation Analyser model K-3002; Kselmed, Grudziadz, Poland) [21].

### The measurement of TT

Human plasma (50 μl) was added to the measuring cuvette and incubated for 1 minute at 37°C on a block heater. The measuring cuvette was transferred to the measuring holes and 100 μl of thrombin was added (final concentration—1 U/ml). The TT was determined coagulometrically using a K-3002 Optic Coagulation Analyser (Kselmed, Grudziadz, Poland) [21].

### The measurement of APTT

Human plasma (50 μl) was added to measuring cuvette and incubated with Dia-PTT liquid (commercial thromboplastin) for 3 minutes at 37°C on a block heater. The measuring cuvette was transferred to the measuring holes and 50 μl of 25 mM CaCl_2_ was added. The APTT was determined coagulometrically using a K-3002 Optic Coagulation Analyser (Kselmed, Grudziadz, Poland) [21].

### Data analysis

In order to eliminate uncertain data, the Q-Dixon test was performed. All the values in this study were expressed as mean ± SD. Obtained results firstly were analysed under the account of normality with Shapiro-Wilk test and equality of variance with Levine test. Statistically significant differences were assessed by applying the ANOVA test, followed by Tukey multiple comparisons test or Kruskal-Wallis test.

## Results

**Fig 1:**
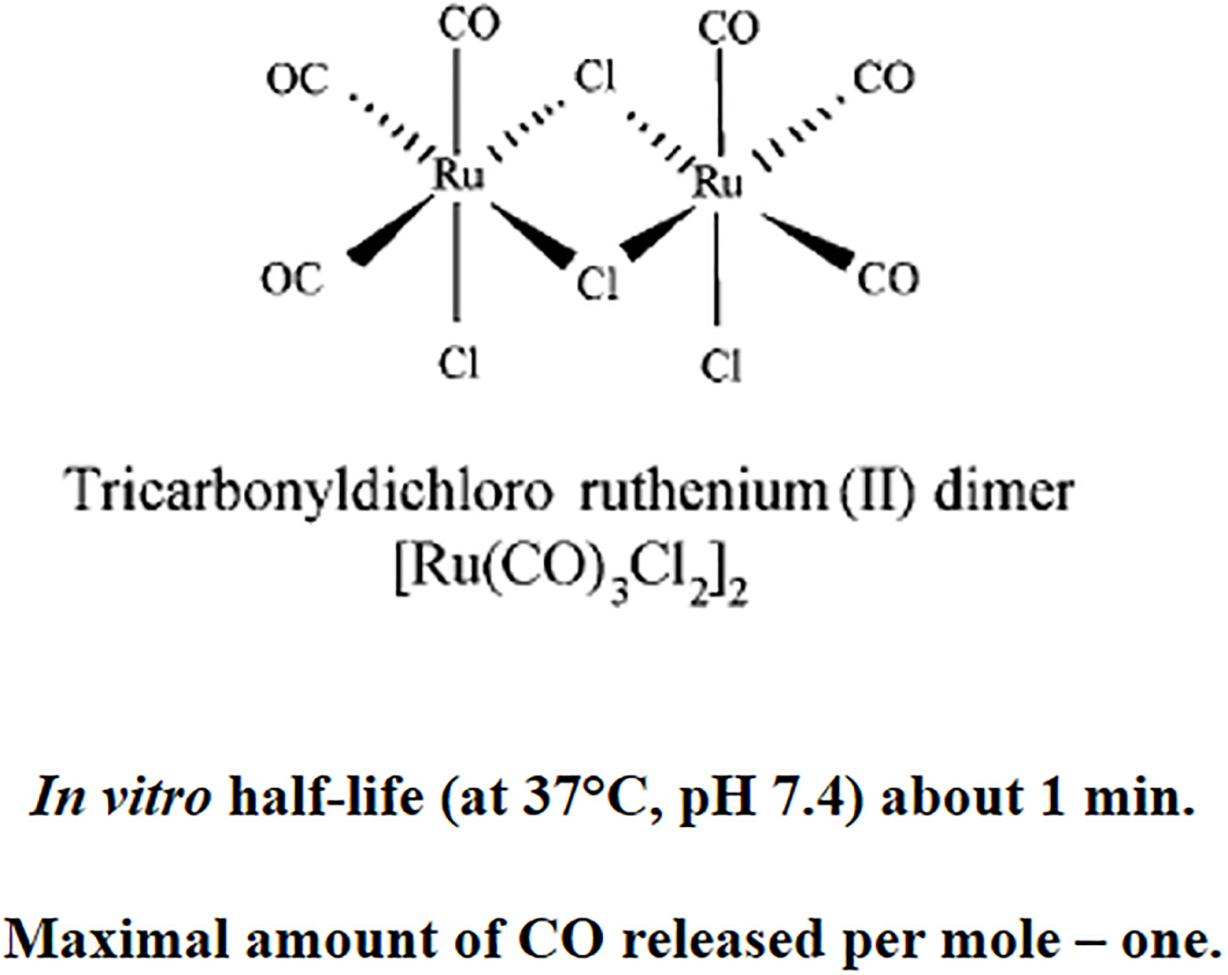
Chemical structure and characteristics of CORM-2 [14; modified].

**Fig 2:**
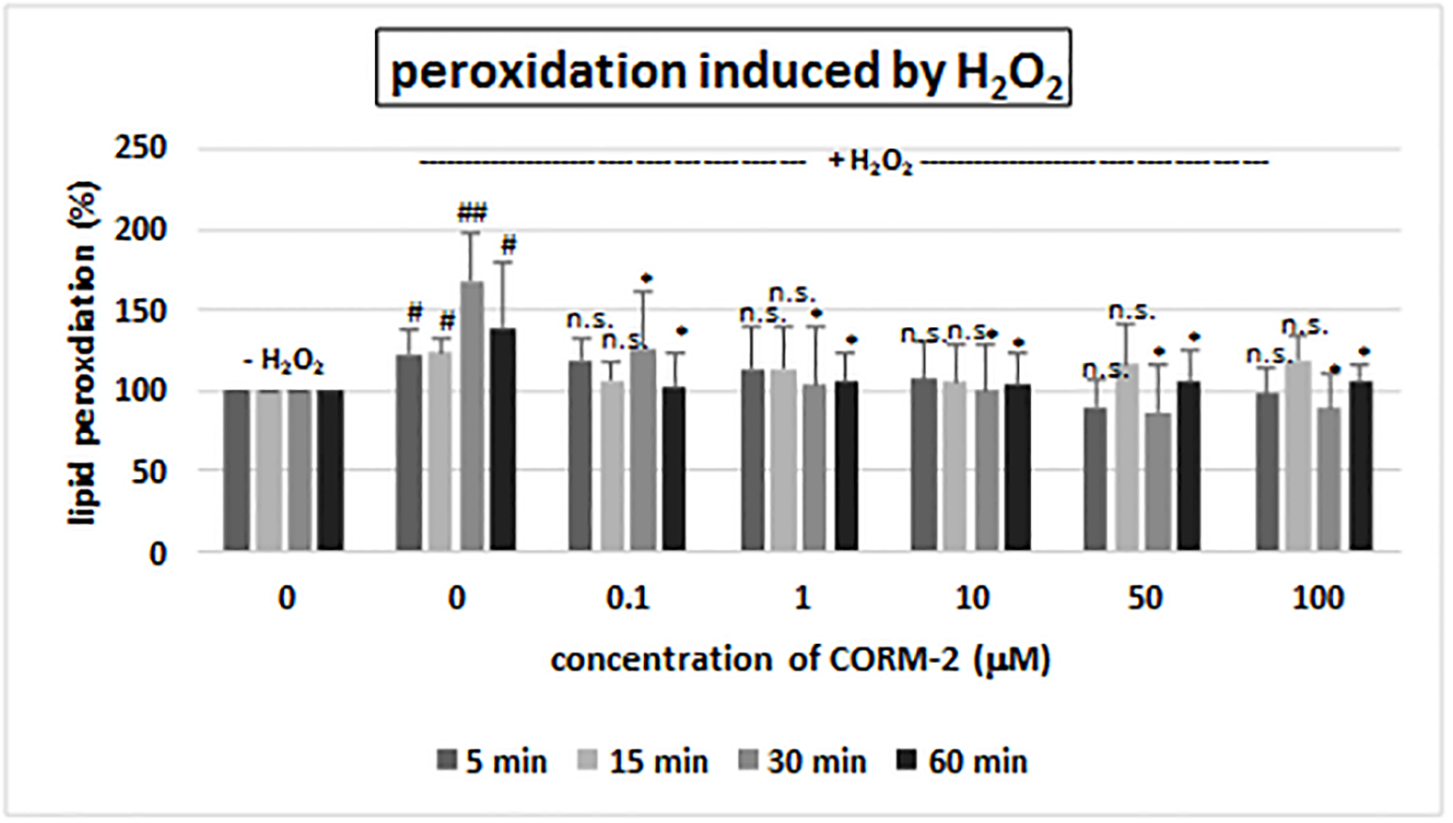
The effect of CORM-2 (0.1–100 μM, incubation time: 5–60 min) on plasma lipid peroxidation induced by H_2_O_2_. Data represents means ± SD of 6 experiments (from different donors). * p<0.05 *vs*. control (+ H_2_O_2_), n.s. p>0.05 *vs*. control (+ H_2_O_2_); # p<0.05 *vs*. control (- H_2_O_2_), ## p<0.02 *vs*. control (- H_2_O_2_).

**Fig 3:**
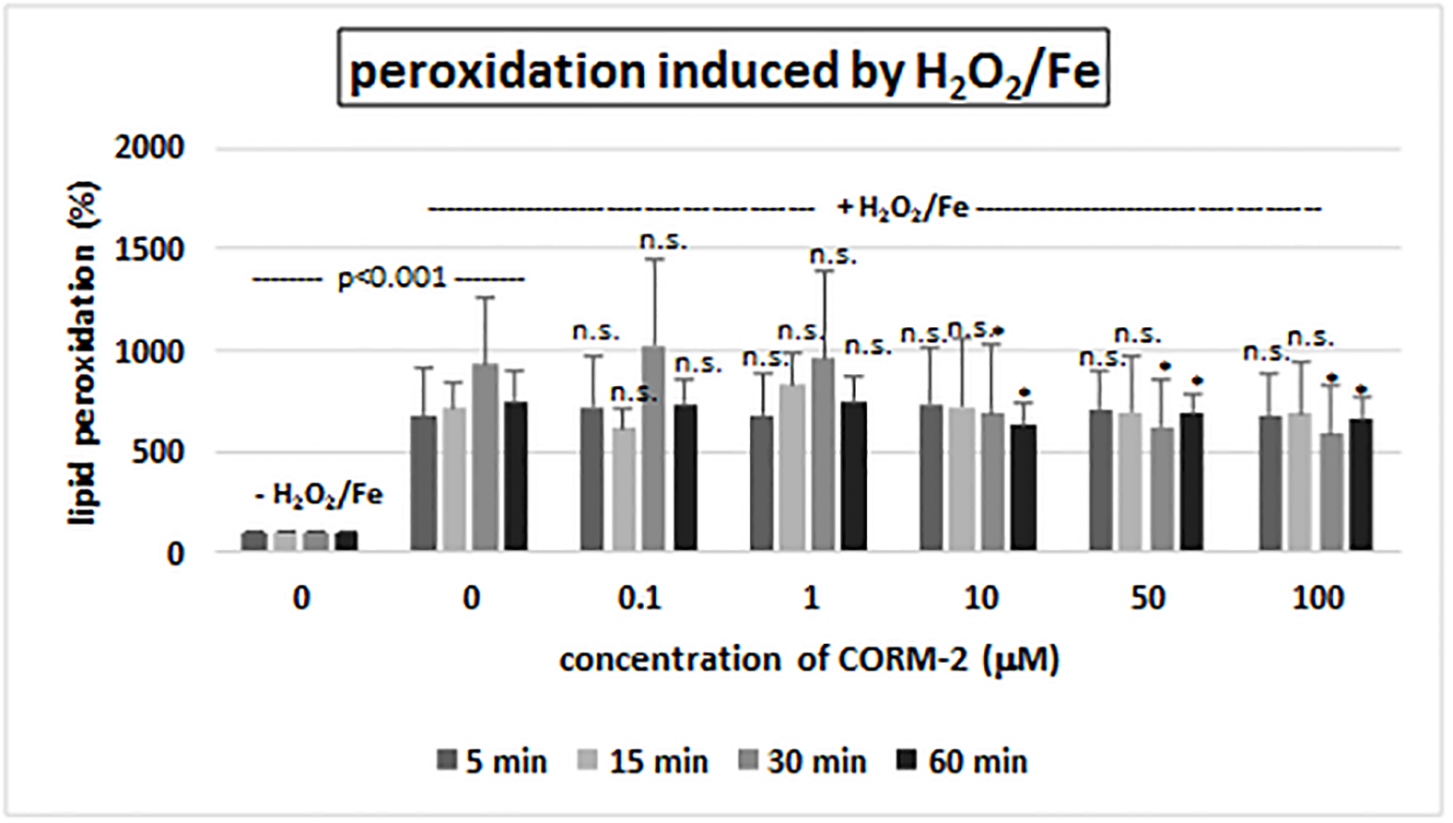
The effect of CORM-2 (0.1–100 μM, incubation time: 5–60 min) on plasma lipid peroxidation induced by H_2_O_2_/Fe. Data represents means ± SD of 6 experiments (from different donors). * p<0.05 *vs*. control (+ H_2_O_2_/Fe), n.s. p>0.05 *vs*. control (+ H_2_O_2_/Fe).

CORM-2 (Fig 1) added to human plasma (treated with H_2_O_2_ or H_2_O_2_/Fe) *in vitro* at concentrations of 0.1–100 μM had different effect on lipid peroxidation (measured by TBARS level) after 5, 15, 30 and 60 minutes of incubation (Figs 2 and 3). The lipid peroxidation induced by H_2_O_2_ was not influenced by CORM-2 at short incubation time (5 and 15 minutes) (Fig 2). However, CORM-2 (at all tested concentrations: 0.1, 1, 10, 50 and 100 μM, and at longer incubation time: 30 and 60 minutes) reduced lipid peroxidation induced by H_2_O_2_ (Fig 2). CORM-2 (at higher concentrations: 10, 50 and 100 μM) after long incubation times (30 and 60 minutes) significantly also reduced lipid peroxidation induced by H_2_O_2_/Fe (Fig 3).

**Fig 4:**
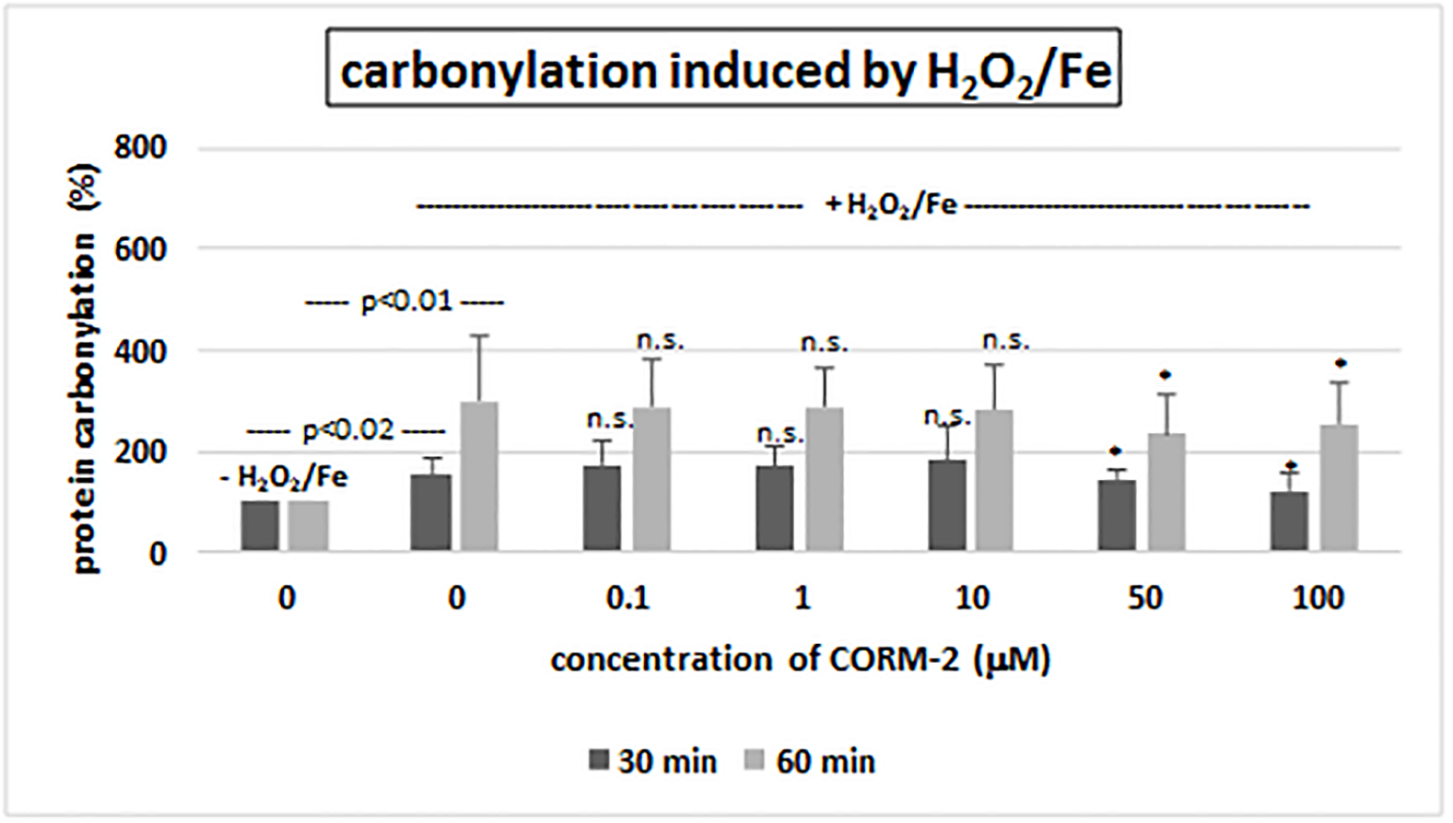
The effect of CORM-2 (0.1–100 μM, incubation time– 30 and 60 min) on carbonyl group formation (plasma protein oxidation) induced by H_2_O_2_/Fe. Data represents means ± SD of 6 experiments (from different donors). * p<0.05 *vs*. control (+ H_2_O_2_/Fe), n.s. p>0.05 *vs*. control (+ H_2_O_2_/Fe).

Although CORM-2 did not change the level of carbonyl groups in plasma proteins when administered without the addition of H_2_O_2_ or H_2_O_2_/Fe (p>0.05) (data are not presented), a significant decrease was observed in the level of carbonyl groups in plasma proteins compared with control—H_2_O_2_/Fe, when human plasma was incubated with the highest tested concentrations of CORM-2–50 and 100 μM and with H_2_O_2_/Fe (Fig 4). In the presence of highest concentration of CORM-2 (100 μM) protein carbonylation was reduced by about 30% (for time incubation– 30 min) and by about 15% (for time incubation– 60 min) (Fig 4).

**Fig 5:**
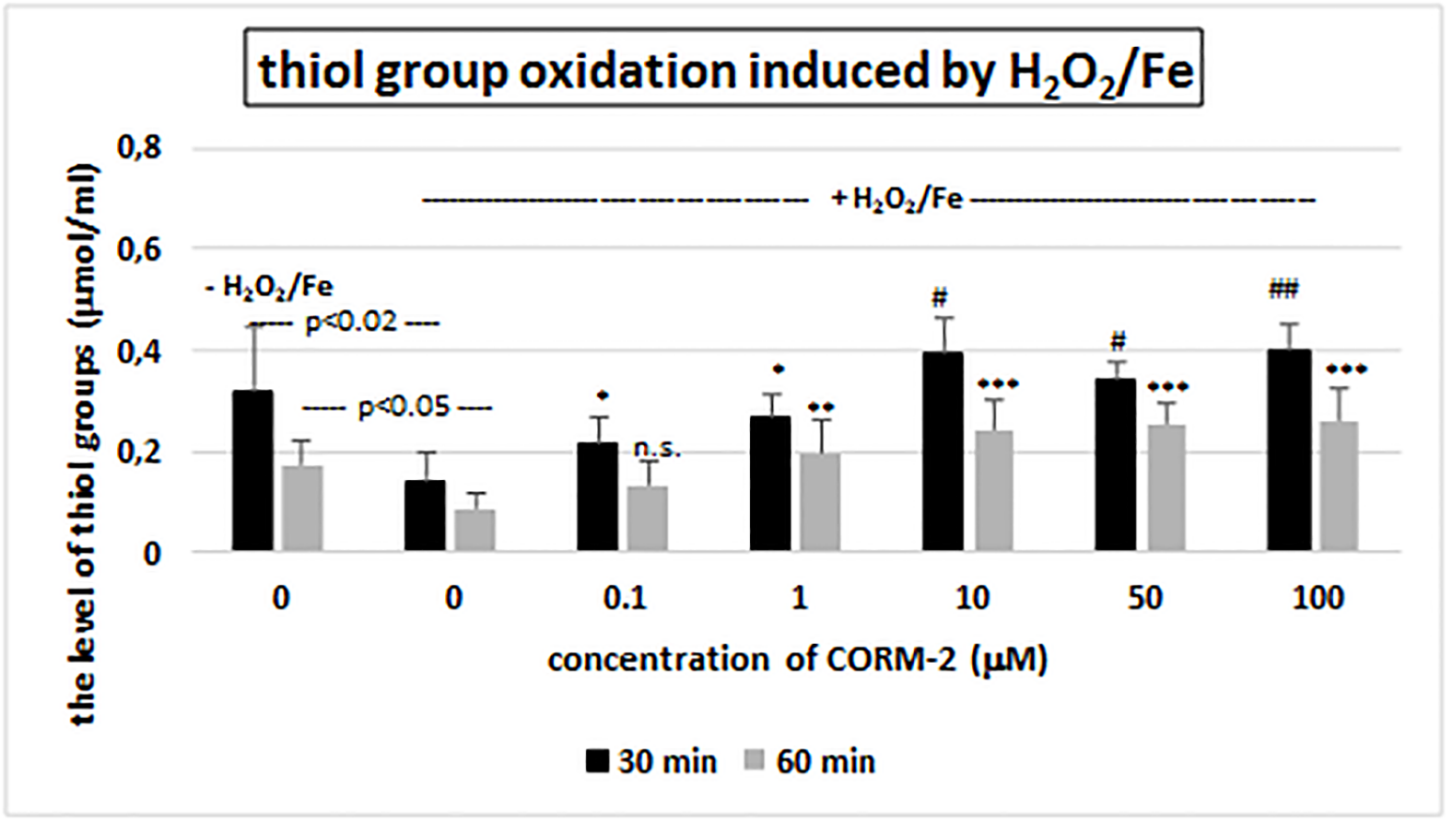
The effect of CORM-2 (0.1–100 μM, incubation time– 30 and 60 min) on the oxidation of protein thiols induced by H_2_O_2_/Fe. Data represents means ± SD of 6 experiments (from different donors). * p<0.05 *vs*. control (+ H_2_O_2_/Fe), ** p<0.01 *vs*. control (+ H_2_O_2_/Fe), *** p<0.0002 *vs*. control (+ H_2_O_2_/Fe), # p<0.0005 *vs*. control (+ H_2_O_2_/Fe), ## p<0.00002 *vs*. control (+ H_2_O_2_/Fe), n.s. p>0.05 *vs*. control (+ H_2_O_2_/Fe).

Another set of experiments focused on the determination of oxidation of plasma protein thiols. We observed that CORM-2 (at all tested concentrations) had strong protector effect on the changes of the level of thiol groups in plasma proteins induced by H_2_O_2_/Fe (Fig 5). The CORM-2 activity was concentration—dependent (Fig 5).

**Fig 6:**
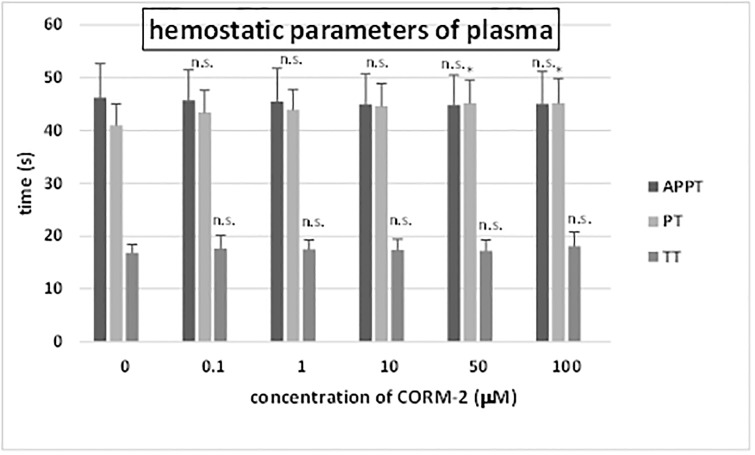
The effect of CORM-2 (0.1–100 μM, incubation time– 30 min) on the hemostatic parameters of human plasma (APPT, PT and TT). Data represents means ± SD of 10 experiments (from different donors). * p<0.05 *vs*. control, n.s. p>0.05 *vs*. control.

Incubation (30 minutes) with CORM-2 resulted in changes of coagulation activity of human plasma (Fig 6). We demonstrated that incubation of plasma with CORM-2 (at high tested concentrations: 50 and 100 μM) significantly prolonged the prothrombin time. The studied also showed that the exposure of plasma to CORM-2 (0.1–100 μM) did not change in the TT and APTT (Fig 6).

**Fig 7:**
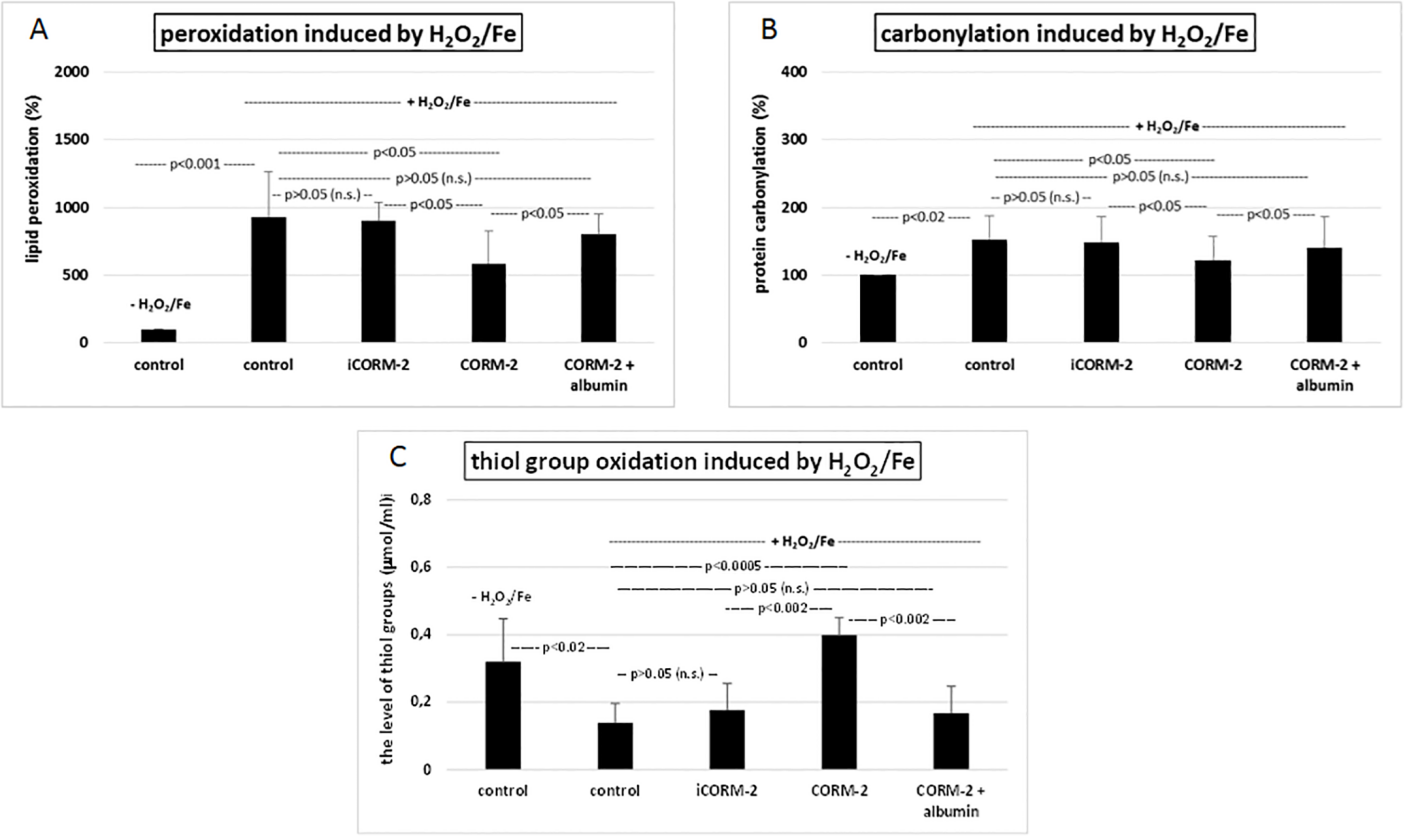
The effect of iCORM-2 (100 μM, incubation time—30 min) or CORM-2 (100 μM, incubation time—30 min) and albumin (10%, incubation time—30 min) on biomarkers of oxidative stress (lipid peroxidation (A), carbonyl group formation (B), oxidation of protein thiols (C)) in plasma treated with H_2_O_2_/Fe. Data represents means ± SD of 5–11 experiments (from different donors).

**Fig 8:**
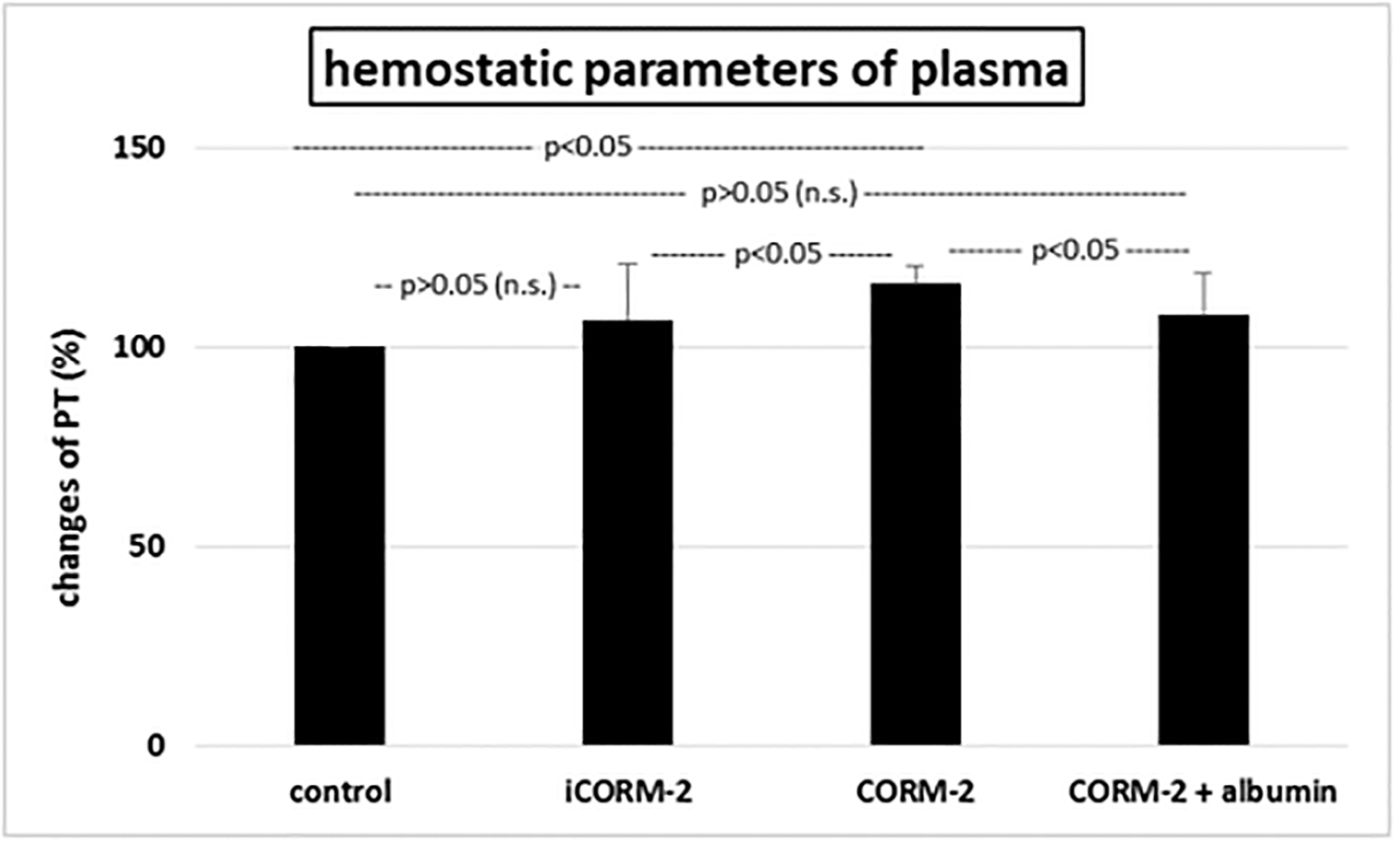
The effect of iCORM-2 (100 μM, incubation time—30 min) or CORM-2 (100 μM, incubation time—30 min) and albumin (10%, incubation time—30 min) on the PT of plasma. In these experiments, the PT in control samples was 45.6 ± 10.2 s. Data represents means ± SD of 5–15 experiments (from different donors).

iCORM-2 had no effect on biomarkers of oxidative stress, which we studied *in vitro* (in compare to control—plasma treated with H_2_O_2_/Fe) (Fig 7). However, 100 μM CORM-2 significantly reduced lipid peroxidation, protein carbonylation and oxidation of protein thiols in plasma treated with H_2_O_2_/Fe (in compare not only to plasma treated only with H_2_O_2_/Fe, but also to plasma treated with iCORM-2 and H_2_O_2_/Fe). The same process was observed in presence of albumin and CORM-2 (Fig 7). In addition, iCORM (100 μM) had no effect on hemostatic parameters of plasma. It did not change the PT (Fig 8) and the TT and APTT (data not shown). Moreover, CORM-2 (100 μM) in presence of albumin did not change in the level of tested hemostatic parameters of plasma (Fig 8).

In control experiments, we observed that DMSO (the solvent of CORM-2) added to plasma at a final concentration <0.05% did not change the level of biomarkers of oxidative stress and tested hemostatic parameters (data not shown).

## Discussion

CORMs are releasing CO in a concentration-dependent manner, modulated by temperature, pH and environment (solvents) [22]. The effects of various donors of CO on different components of hemostasis, i.e. blood platelets, coagulation and fibrinolysis process may be manifold due to their pleiotropic character. However, there are only a few experiments addressing the anti-caoagulatory, pro-coagulatory and anti-platelet effects of CORMs [10, 12, 23, 24]. Liu et al. [10] have observed that CORM-2 inhibits the abnormal activation of blood platelets stimulated by endotoxin. The results of Nielsen and Pretorius [24] have demonstrated that CO derived from CORM-2 interacts with coagulation and fibrinolytic process, resulting in thrombi that begin to form more quickly, grow faster, become stronger, and are more resistant to lysis. In these experiments, the effects of CORMs on coagulation were determined by thrombelastography using either tissue factor or celite activation. The same author have noted the effects of six *Agkistrodon* species’ venoms on plasmatic coagulation, with iron and CORM-2 addition attenuating the degradation of coagulation in a species—specific manner [25]. In addition, CO and iron have modulated plasmatic coagulation in Alzheimer’s disease [7]. Results of Motterlini et al. [26] have indicated that CORMs, including CORM-2 cause vasodilation in precontracted rat aortic rings, attenuate coronary vasoconstriction in hearts *ex vivo*, and reduce acute hypertension *in vivo*.

The present study provides more information on the biological property of CORM-2 in human plasma. First, the results of our studies showed that exogenous CO (as CORM-2) causes changes in hemostasis (determined coagulametrically); at concentrations of 50 and 100 μM prolonges the prothrombin time, which is a measure of the efficiency of extrinsic coagulation system. Prolongation of prothrombin time by CORM-2 can attest to its effect on prothrombin activity or clotting factors V, VII, and X. This obtained results indicate the anti-coagulant activities of CORM-2 in model system *in vitro*. We also demonstrated changes in lipid peroxidation, formation of carbonyl groups and oxidation of thiol groups in human plasma treated with CORM-2. Our present experiments have demonstrated that in human plasma, the properties of CORM-2 depend on very often its concentration and incubation time. Our work was designed to estimate the antioxidative or prooxidative effects of CORM-2 on human plasma lipid and protein changes stimulated by two biological oxidants: H_2_O_2_ and H_2_O_2_/Fe (*in vitro*). In our present experiments, we used two oxidative conditions, namely (I) H_2_O_2_ and (II) H_2_O_2_/Fe, because the concentration of Fe is low in isolated human plasma, and the level of markers of oxidative stress (i.e. TBARS) is also low. However, when we added Fe to plasma, we observed higher level of these biomarkers of oxidative stress. In addition, some authors believe that some complexes of iron ions (i.e. EDTA) may also react with H_2_O_2_ to form OH [27,28]. The results from our experiments show that CORM-2 (at the highest tested concentrations) had an inhibitory action on H_2_O_2_ or H_2_O_2_/Fe—induced oxidation in plasma. These results are consistent with other studies on the role of CO donors in protecting against oxidative stress, i.e. studies of Tsai et al. [8] demonstrated that CORM-2 inhibits angiotensin II-induced human aortic smooth muscle cells migration through inactivation of NADPH oxidase/reactive oxygen species generation. Babu et al. [14] also noted that CORM-2 shows antioxidant property in murine intestinal epithelial MODE-K cells under oxidative stress (induced by H_2_0_2_). Moreover, it is very important that our used concentrations of CORM-2 in our model *in vitro* are consistent with other studies [8, 11, 14, 25, 29], i.e. Tsai et al. [8] observed that 50 μM CORM-2 has antioxidant activity. Results of Srisook et al. [29] have also demonstrated that 50 μM CORM-2 inhibits the production of superoxide anion (O_2_^-·^) and nitric oxide (NO^·^) in macrophages stimulated by bacterial lipopolysaccharide (LPS) *in vitro*. The amount of CORM-2 used *in vitro* in the present experiment and by other authors is significantly less than has been administrated *in vivo* [30, 31]. In addition, our present results show that CORM-2 especially at high tested concentrations (10, 50 and 100 μM) could be used as an antioxidant (when the oxidative stress is stimulated not only by H_2_O_2_, but also by a hydroxyl radical donor—H_2_O_2_/Fe).

It is very important that in cardiovascular system, CO does not work often in isolation, but it may interact with reactive oxygens species or reactive nitrogen species, i.e. nitric oxide, which may be secondary messengers in various biological systems. The obtained results indicate not only the antioxidant activities, but also anticoagulant properties of CORM-2 *in vitro*. Therefore, we suppose that CORM-2 may be promising compound to prevent thrombosis in pathological processes where plasma procoagulant activity and oxidative stress are observed. However, the mechanism(s) of CORM-2 action on hemostasis has not yet well know. Recognition of CO donors, including CORM-2 as both an antioxidant and a prooxidant may enhance the treatment of various diseases associated with oxidative stress. Moreover, further experiments are need to gain a more complete understanding of the dual nature of the procoagulant and anticoagulant of CORMs. It may be especially very important in cardiovascular diseases, in which oxidative stress may modulate various elements of hemostasis. The further experiments should be done to establish the effects of CORM-2 (as an antioxidant) on activity of different elements of hemostasis, including blood platelets.

## Supporting information

S1 Table The effect of CORM-2 (0.1–100 μM, incubation time: 5–60 min) on plasma lipid peroxidation induced by H_2_O_2_. (TIF)

S2 Table The effect of CORM-2 (0.1–100 μM, incubation time: 5–60 min) on plasma lipid peroxidation induced by H_2_O_2_/Fe. (TIF)

S3 Table The effect of CORM-2 (0.1–100 μM, incubation time—30 and 60 min) on carbonyl group formation (plasma protein oxidation) induced by H_2_O_2_/Fe. (TIF)

S4 Table The effect of CORM-2 (0.1–100 μM, incubation time—30 and 60 min) on the oxidation of protein thiols induced by H_2_O_2_/Fe. (TIF)

S5 Table The effect of CORM-2 (0.1–100 μM, incubation time—30 min) on the hemostatic parameters of human plasma (APPT, PT and TT). (TIF)

S6 Table The effect of iCORM-2 (100 μM, incubation time—30 min) or CORM-2 (100 μM, incubation time—30 min) and albumin (10%, incubation time—30 min) on biomarkers of oxidative stress (lipid peroxidation, carbonyl group formation, oxidation of protein thiols) in plasma treated with H_2_O_2_/Fe. (TIF)

S7 Table The effect of iCORM-2 (100 μM, incubation time—30 min) or CORM-2 (100 μM, incubation time—30 min) and albumin (10%, incubation time—30 min) on the PT of plasma. (TIF)
