# Supplementary figures and images for: The role of CORM-2 as a modulator of oxidative stress and hemostatic parameters of human plasma in vitro
Source: PLoS One. 2017 Sep 26;12(9):e0184787. doi: 10.1371/journal.pone.0184787 (PMC5614530; doi:10.1371/journal.pone.0184787)

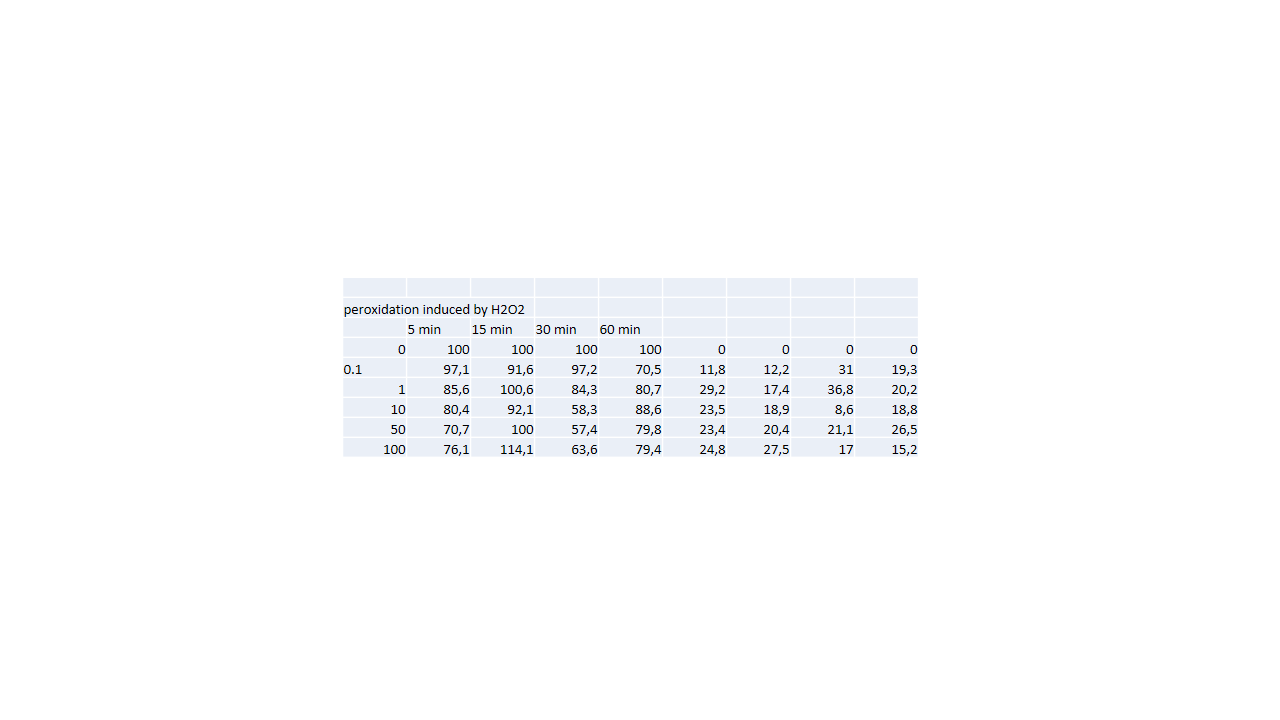

Supplement: S1 Table — (TIF) [file pone.0184787.s001.TIF]

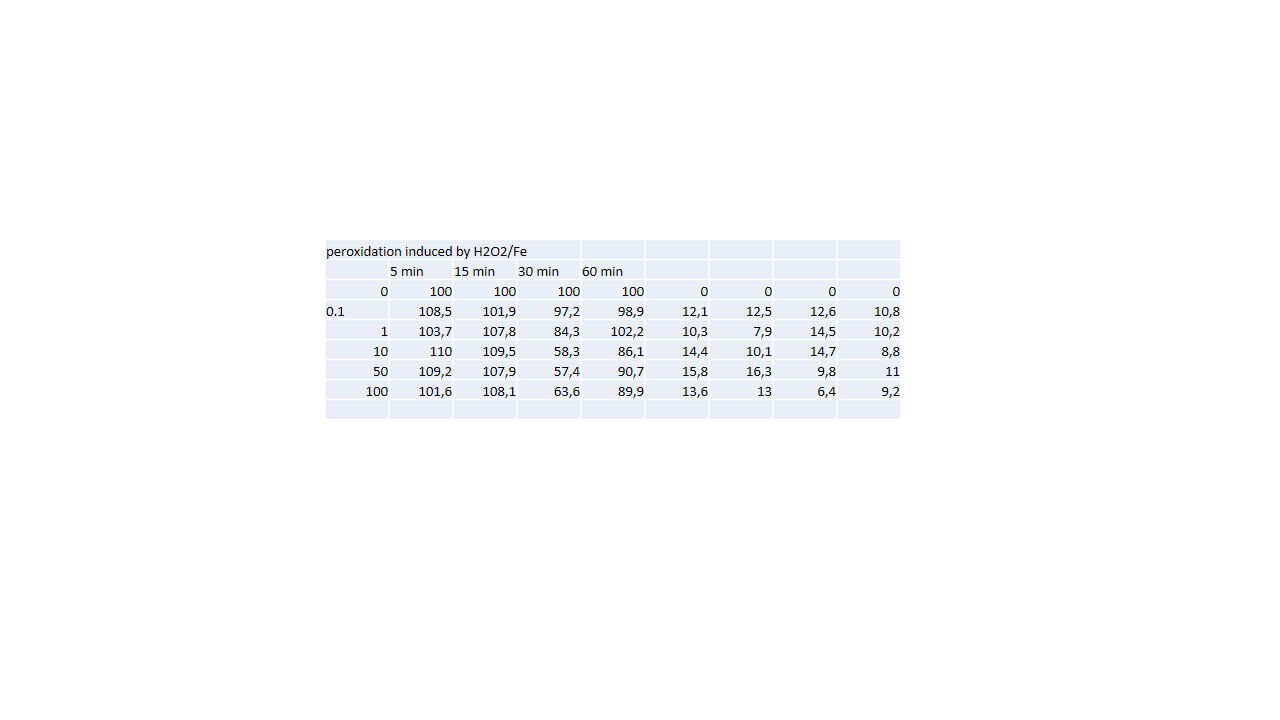

Supplement: S2 Table — (TIF) [file pone.0184787.s002.TIF]

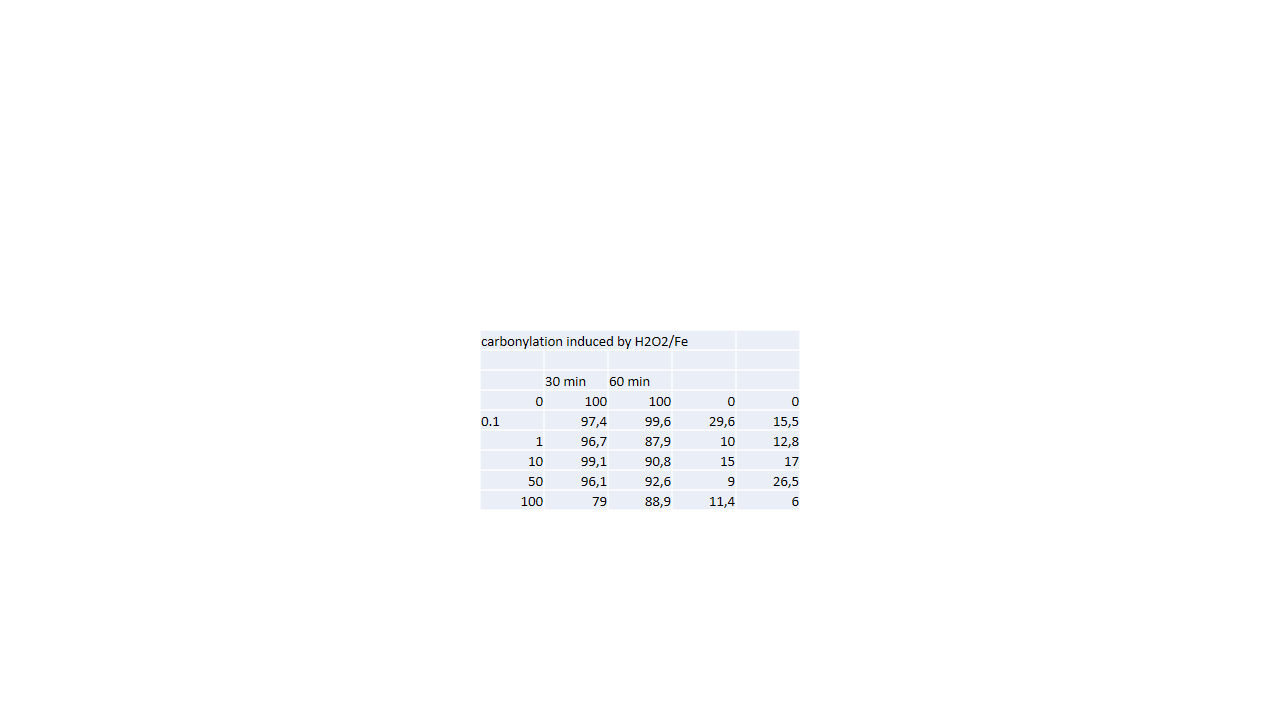

Supplement: S3 Table — (TIF) [file pone.0184787.s003.TIF]

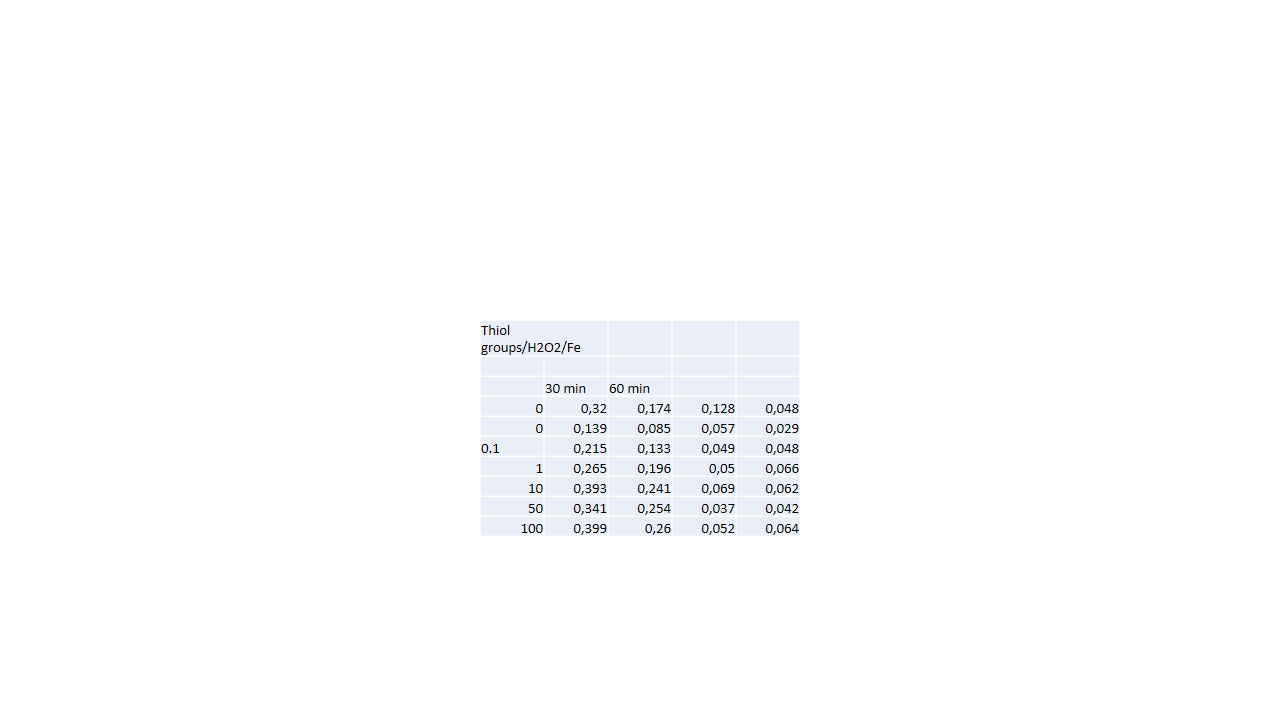

Supplement: S4 Table — (TIF) [file pone.0184787.s004.TIF]

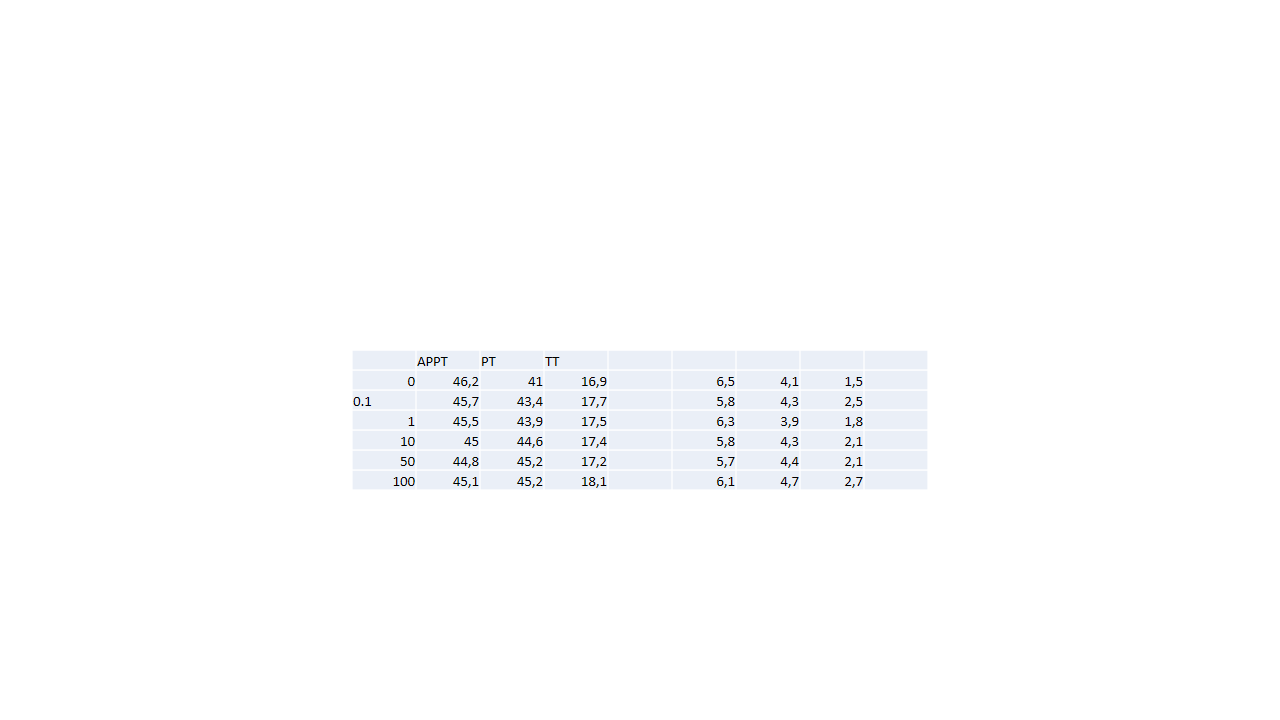

Supplement: S5 Table — (TIF) [file pone.0184787.s005.TIF]

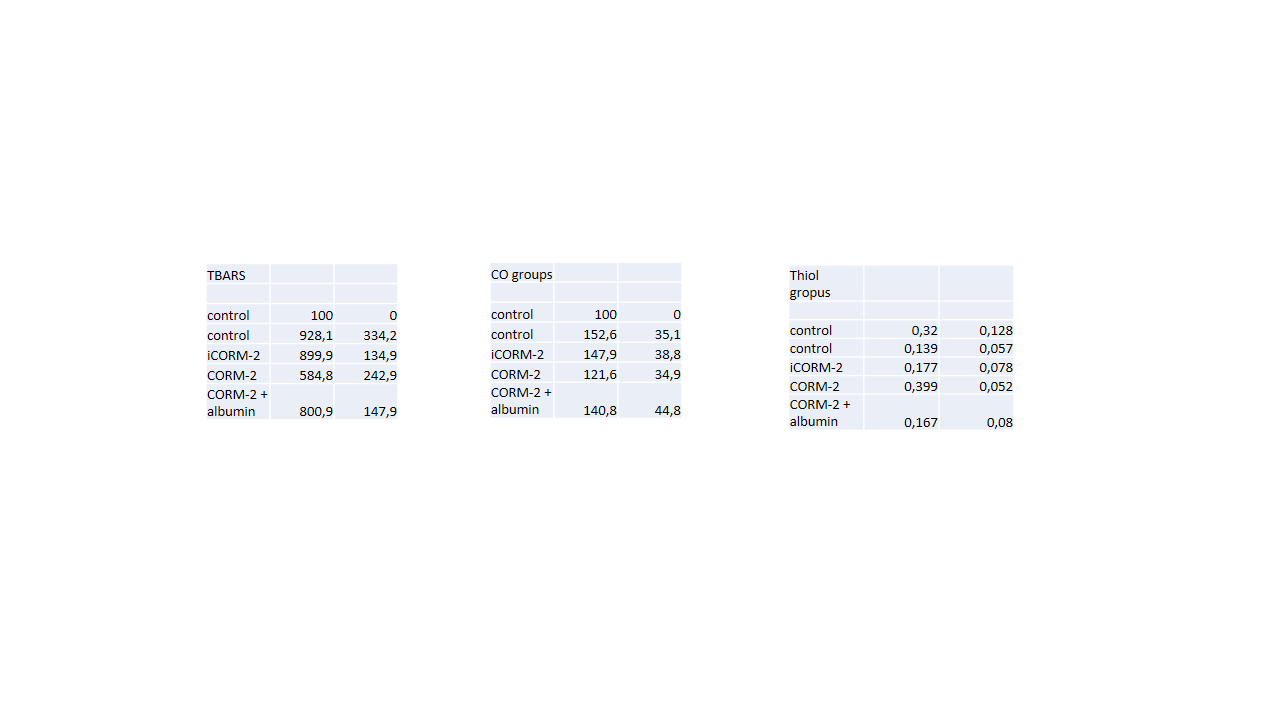

Supplement: S6 Table — (TIF) [file pone.0184787.s006.TIF]

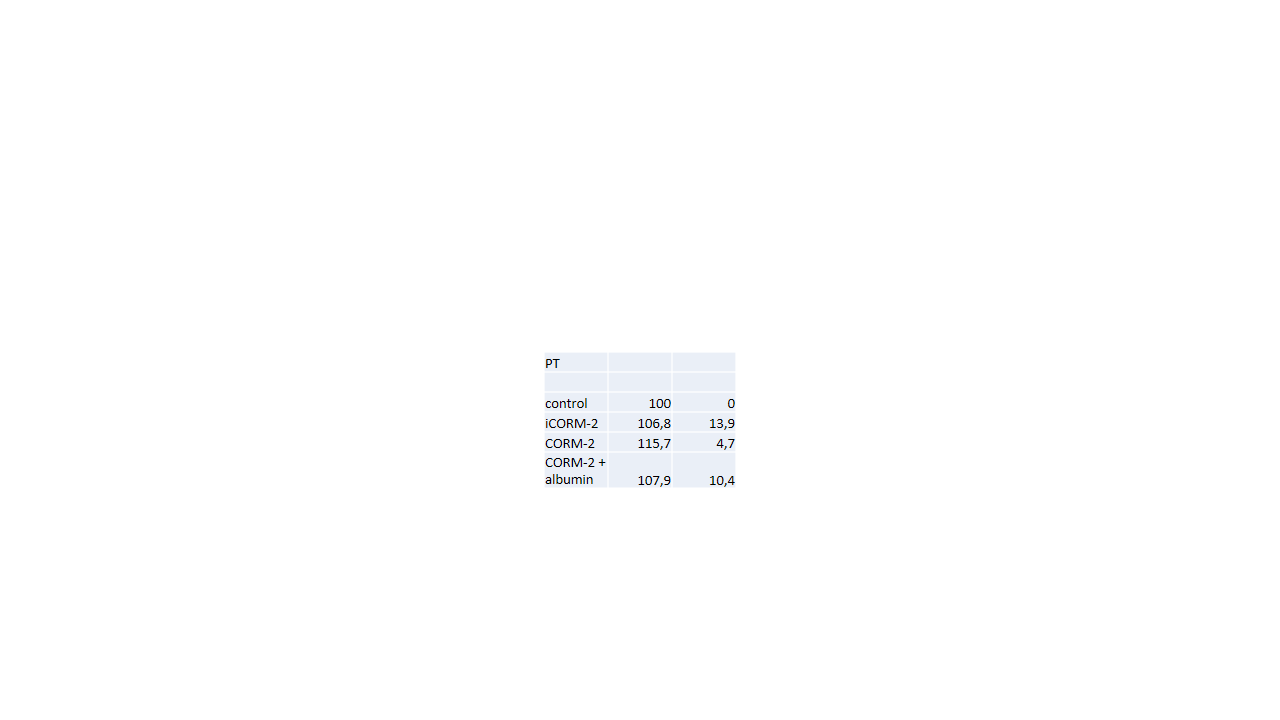

Supplement: S7 Table — (TIF) [file pone.0184787.s007.TIF]
